# Incidence of Paradoxical Tuberculosis-Associated Immune Reconstitution Inflammatory Syndrome and Impact on Patient Outcome

**DOI:** 10.1371/journal.pone.0084585

**Published:** 2013-12-18

**Authors:** Maryline Bonnet, Elisabeth Baudin, Ilesh V. Jani, Elizabete Nunes, François Verhoustraten, Alexandra Calmy, Rui Bastos, Nilesh B. Bhatt, Christophe Michon

**Affiliations:** 1 Epicentre, Paris, France; 2 Instituto Nacional de Saúde, Maputo, Mozambique; 3 Hospital Central, Maputo, Mozambique; 4 Médecins sans Frontières, Geneva, Switzerland; 5 Geneva University Hospital, Geneva, Switzerland; 6 Annecy Hospital, Annecy, France; Temple University School of Medicine, United States of America

## Abstract

**Objectives and Design:**

We used data from a randomized trial of HIV-tuberculosis co-infected patients in Mozambique to determine the incidence and predictors of paradoxical tuberculosis-associated immune reconstitution inflammatory syndrome (IRIS) occurring within 12 weeks of starting antiretroviral therapy, and to evaluate its association with patient outcome at 48 weeks.

**Methods:**

HIV-tuberculosis co-infected and antiretroviral therapy-naïve adults with less than 250 CD4/mm3 were randomized to a nevirapine or efavirenz-based antiretroviral therapy initiated 4 to 6 weeks after starting tuberculosis treatment, and were then followed for 48 weeks. Tuberculosis cases were diagnosed using WHO guidelines, and tuberculosis-IRIS by case definitions of the International Network for the Study of HIV-associated IRIS.

**Results:**

The 573 HIV-tuberculosis co-infected patients who initiated antiretroviral therapy had a median CD4 count of 92 cells/mm^3^ and HIV-1 RNA of 5.6 log_10_ copies/mL. Mortality at week 48 was 6.1% (35/573). Fifty-three (9.2%) patients presented a tuberculosis-IRIS within 12 weeks of starting antiretroviral therapy. Being female and having a low CD4 count, high HIV-1 RNA load, low body mass index and smear-positive pulmonary tuberculosis were independently associated with tuberculosis-IRIS. After adjustment for baseline body mass index, CD4 count and hemoglobin, occurrence of tuberculosis-IRIS was independently associated with 48-week mortality (aOR 2.72 95%CI 1.14-6.54). Immunological and HIV-1 virological responses and tuberculosis treatment outcomes were not different between patients with and without tuberculosis-IRIS.

**Conclusion:**

In this large prospective cohort, tuberculosis-IRIS occurrence within 12 weeks of starting antiretroviral therapy was independently associated with the mortality of HIV-tuberculosis co-infected patients at 48 weeks post antiretroviral therapy initiation.

## Introduction

Tuberculosis (TB) remains the most common opportunistic infection in patients living with HIV/AIDS and one of the main causes of death before and during the first months of antiretroviral therapy (ART)[1]. Mortality is driven by too many cases of undiagnosed TB in advanced HIV patients not on ART, reflecting the frequent lack of systematic TB screening prior to ART initiation, absence of effective TB diagnostic tools, and late initiation of ART [2-4]. Recent studies have shown significant reduction in mortality when ART is initiated within two weeks of starting anti-TB treatment in severely immunocompromised patients [5-8]. However, early ART initiation increases the risk of immune reconstitution inflammatory syndrome (IRIS) [7]. 

IRIS results from the rapid restoration of pathogen-specific immune responses to opportunistic infections, which in turn causes either worsening of an already-diagnosed infection (paradoxical TB-IRIS) or presentation of a previously subclinical infection (unmasking IRIS) [9]. In a meta-analysis performed in 2010, paradoxical TB-associated IRIS was reported in 15.7% (95%CI 9.7-24.5) of HIV-infected patients treated for TB, with death reported for 3.2% (95%CI 0.7-9.2) of those with TB-IRIS [10]. Low CD4 cell count at ART initiation and short time interval between starting TB therapy and ART were the best predictors of paradoxical TB-associated IRIS [11-13]. Despite a standardized case definition proposed in 2008 by the International Network for the Study of HIV-associated IRIS (INSHI) for high HIV burden and resource-limited countries, the diagnosis of paradoxical TB-associated IRIS still remains empirical and challenging due to the lack of specific diagnostic tests [13]. 

While several studies have evaluated the incidence and predictors of paradoxical TB-associated IRIS (TB-IRIS), very few have assessed the association between TB-IRIS occurrence and the long-term outcomes of HIV-TB co-infected patients in terms of mortality, HIV and/or TB treatment. Here we report an analysis of data from a large randomized trial of HIV-TB co-infected patients in Mozambique to determine the incidence and predictors of TB-IRIS and to assess the association between the occurrence of TB-IRIS and 48-week patient outcomes. 

## Material and Methods

### Ethics statement

Ethical approval of the study protocol was obtained from the Comité Nacional de Bio-Ética para a Saúde (Maputo, Mozambique); Médecins Sans Frontières Ethics Review Board (Zurich, Switzerland); Comité de Protection des Personnes (Saint Germain-en-Laye, France) and the Columbia University Institutional Review Board (New York, United States). All participants gave signed informed consent.

The ANRS 12146 – CARINEMO trial was an open label, randomized, phase III non-inferiority trial which compared the efficacy and safety of ART regimens based on nevirapine (without a leading dose) *vs* efavirenz in HIV-TB co-infected patients receiving rifampicin as part of their TB treatment regimen. The trial was registered with ClinicalTrials.gov, number NCT00495326)[14]. ART-naïve HIV-infected patients, aged 18 years or older and with active TB, were identified at the TB- or HIV outpatient clinics of three health centres in Maputo city, Mozambique. Participants were eligible for enrolment in the study if they had a Karnofsky score of 60% or greater, CD4 count below 250 cells/mm^3^, negative urine pregnancy test for women, alanine aminotransferase (ALAT), total bilirubin levels below 5 times the upper normal limit (UNL) (grade <3), and absence of any serious clinical symptom or laboratory results (grade 4). Patients were randomized to nevirapine or efavirenz regimens in a 1:1 ratio 4-6 weeks after starting TB treatment.

TB diagnosis was based on WHO guidelines. Pulmonary TB was diagnosed by microscopic sputum examinations, chest X-ray and absence of a favorable response to antibiotics in patients with smear-negative sputum [15]. Extra-pulmonary TB was diagnosed by cyto-histopathology, clinical presentation, chest X-ray assessment and ultrasonography, as appropriate. Because the Mozambican national TB program does not routinely use *Mycobacterium tuberculosis* culture to diagnose pulmonary TB, culture was performed only on specimens collected at the first screening visit in the trial, i.e., approximately two weeks after TB treatment initiation. Indeed, most patients were referred to the trial sites only after starting TB treatment. At this first visit two sputum specimens were collected and then shipped to the reference laboratory at the Institute of Tropical Medicine (Antwerp, Belgium) for culture and drug susceptibility testing. 

All patients received the standard national anti-TB therapy (a fixed-dose combination of isoniazid, rifampicin, ethambutol and pyrazinamide) for the first 2 months, followed by isoniazid and rifampicin for the subsequent 4 months. ART was started either with a fixed-dose combination of nevirapine, lamivudine and stavudine (Triomune®) or with efavirenz plus lamivudine and stavudine. Patient counselling, which included psychosocial support and education on adherence to ART and TB therapy, preceded ART initiation. Nevirapine was initiated without a leading dose (400 mg/day). In August 2010 stavudine was replaced by zidovudine, to comply with revised national ART treatment recommendations. All patients received pyridoxine (50 mg qd) during TB treatment and trimethoprim-sulfamethoxazole (960 mg qd) for opportunistic infections prophylaxis. 

Clinical examination, isoniazid urine test and pregnancy test for women were performed weekly and for the first 8 weeks after starting ART. ALAT and bilirubin were performed at weeks 2, 4, 6, 12, 16, 20, 24, 36 and 48 weeks. Plasma HIV-1 RNA was measured at ART initiation and then at weeks 12, 24, 36 and 48. CD4 cell count was assessed at baseline and at 24 and 48 weeks. At each follow-up visit, adherence to both ART and TB treatment was monitored by study staff using an analog visual scale, questionnaire and pill count. In addition, the presence of isoniazid metabolites in the urine was assessed using the INH urine test BBL™ Taxo™ INH test (Becton Dickinson, USA).

Diagnosis of paradoxical TB-associated IRIS was based on the INSHI 2008 case definition, which includes three components: 1) Diagnosis of TB before ART initiation, and clinical improvement under TB treatment; 2) Onset of TB-associated IRIS manifestations within 3 months of ART initiation, with at least one major criterion (new or enlarging lymph nodes, cold abscesses, or other focal tissue involvement; new or worsening radiological features of TB, CNS TB or serositis) or two minor clinical criteria (new or worsening of constitutional symptoms, respiratory symptoms, abdominal pain accompanied by peritonitis, hepatomegaly, splenomegaly, or abdominal adenopathy) using systematic physical examination together with radiography, ultrasonography, cytology of fluids and laboratory investigations when appropriate; and 3) Exclusion of alternative explanations for clinical deterioration, if possible (e.g., TB treatment failure due to drug resistance, poor adherence to TB treatment, another opportunistic infection or neoplasm, or drug toxicity or reaction)[13]. Other potential causes of IRIS such as cryptococcosis meningitis, dermatomal or multidermatomal zoster, pneumocystic pulmonary infection, hepatitis B flare, Kaposi sarcoma occurring within 12 weeks after starting ART were also reported.

Upon suspicion of TB-IRIS, clinicians recorded patients’ information in a specific TB-IRIS study form. At the end of the study, all forms and patient files were reviewed by a TB-IRIS review committee (CM, MB, NB). To identify possible missed pauci-symptomatic IRIS, the committee also reviewed clinical files of patients presenting within 12 weeks of starting ART and exhibiting signs and symptoms that could fit the definition of TB-IRIS within 12 weeks after starting ART. 

Any patient with worsening clinical status was admitted to a referral hospital, the Hospital Central de Maputo. Files of patient who died were reviewed by a death review committee (NB, RB) to identify the cause of death or to validate the autopsy report, when available. 

### Statistical analysis

The number of participants needed for the main CARINEMO trial (N=573) was calculated based on the objective of testing the non-inferiority of nevirapine in terms of 48-week virological efficacy. The cumulative incidence of TB-IRIS was calculated as the percentage of all patients who developed a TB-IRIS during their first 12 weeks on ART. Patient characteristics were compared between groups of patients with and without TB-IRIS. Further comparisons were performed between patients who presented an IRIS within the first 4 weeks of starting ART and those who presented an IRIS at 4-12 weeks. Predictors of TB-IRIS were identified among patients’ baseline characteristics (age, sex, body mass index (BMI), ART regimen, type of TB, CD4 count, HIV-1 RNA viral load, time interval between starting TB treatment and ART initiation) using univariate and multivariate analysis with logistic regression model. BMI (by reduction of one Kg/m^2^), CD4 count (by reduction of 50 CD4/mm^3^) and HIV-1 RNA (by increase of one log_10_ copies/mL) were included as continuous variables in the model. Factors with an association showing a p-value <0.20 were selected for the multivariate analysis.

Our analysis compared the proportion of patients with a 12-week HIV-1 RNA viral load reduced by at least one log_10_ copies/mL compared to their baseline viral load, and with undetectable viral load (< 50 copies/mL) after 24 and 48 weeks, in patients with and without TB-IRIS. We also compared the median increase of CD4 count after 24 and 48 weeks, and the proportion of patients with TB treatment success at the end of the treatment (cured and treatment completed, and without TB relapse for the subsequent 24 weeks).

Survival analysis using Kaplan Meier estimates was performed separately for patients with and without TB-IRIS. Patients were censored at the date of death, premature follow-up discontinuation (trial withdrawal or lost to follow-up) or at the end of the trial follow-up (48 weeks). The association between the occurrence of a TB-IRIS and 48-week post-ART mortality was assessed after adjusting for any associated factor among patients’ baseline characteristics and for patients’ adherence to ART, using a logistic regression model. For such analysis, we used the cumulative adherence rate for the duration of follow-up in the trial, dividing adherence into two categories (< and > 80% adherence). Data were analyzed with Stata v.12.1 (StataCorp LP, College Station, TX, USA). Statistical comparisons were performed using Chi^2^, Fisher exact and Wilcoxon signed rank tests, as appropriate.

## Results

### Study population

**Figure 1:**
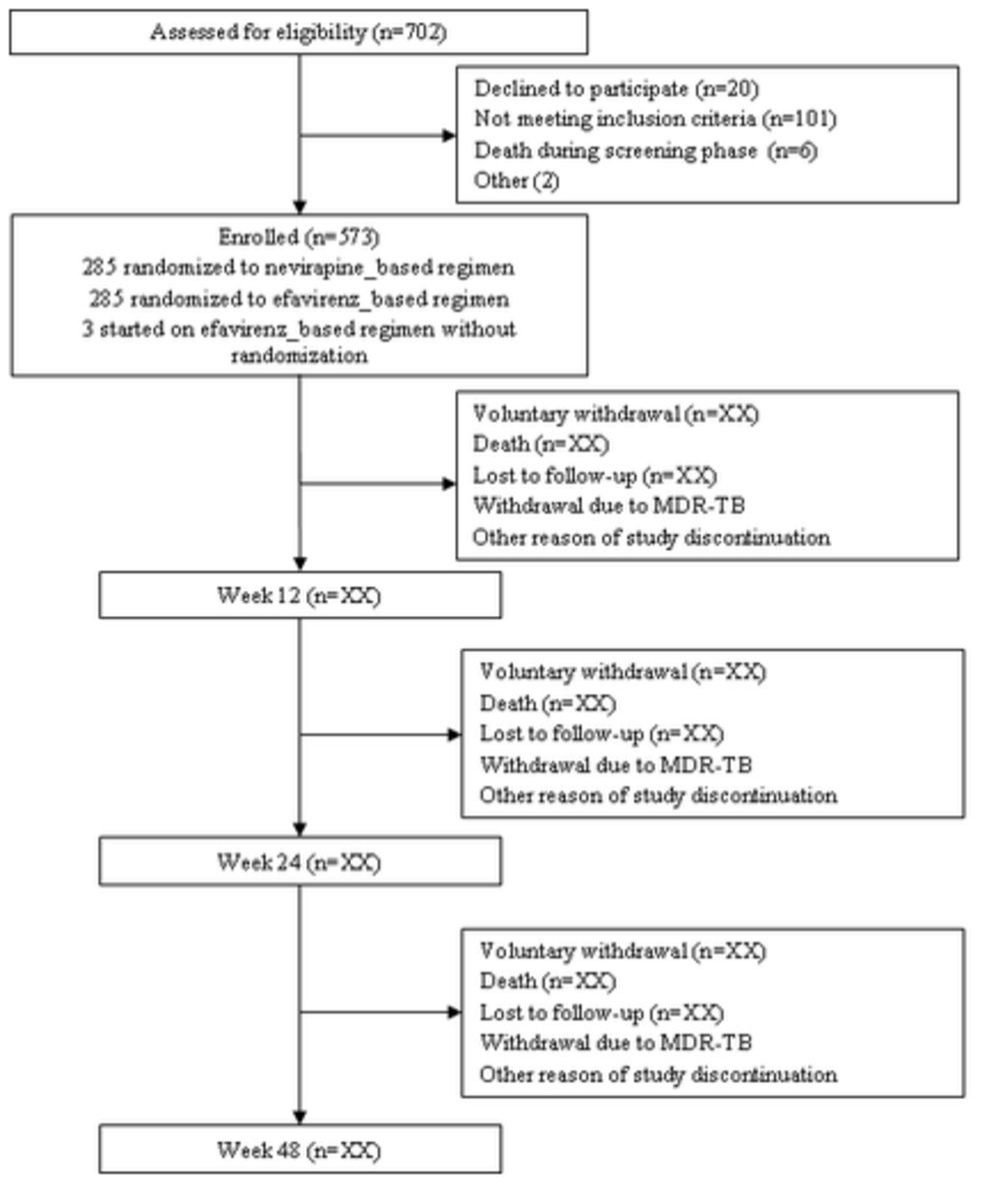
Study profile.

Between October 2007 and March 2010, 720 TB-HIV co-infected patients were screened, of whom 573 (79.6%) were then enrolled in the CARINEMO trial (Figure 1). Six patients died during the screening period, five due to TB (one disseminated TB, one pleural TB and three pulmonary TB) and one due to severe anemia. Of the 573 enrolled patients, 457 (79.8%) completed the 48-week study follow-up, 35 (6.1%) died, 12 (2.1%) were lost to follow-up, and 49 (8.5%) discontinued the study for other reasons (Figure 1). Nevirapine- or efavirenz-based ART regimens were prescribed for 285 and 288 patients, respectively. Patients’ baseline characteristics are presented in Table 1.

**Table 1:** Patient characteristics at ART initiation.

		**Total N=573**	**IRIS N=53**	**No IRIS N=520**
**Age** (years), median [IQR]		33 [29-40]	31 [27-38]	33[29-41]
**Female**, n (%)		212 (40.8)	29 (54.7)	241 (42.1)
Clinical characteristics				
	Body Mass Index (Kg/m^2^), median [IQR]	18.8 [17.3-20.3]	17.9 [16.8-19.2]	18.9 [17.3-20.3]
	Karnofsky score < 80, n (%)	60 (10.5)	7 (15.2)	53 (10.1)
Type of tuberculosis				
Pulmonary Tuberculosis, n (%)				
	Smear-positive pulmonary tuberculosis	220 (38.4)	29 (54.7)	191 (36.7)
	Smear-negative pulmonary tuberculosis	194 (33.9)	18 (34)	176 (33.8)
	Smear missing PT	30 (5.2)	0 (0)	30 (5.8)
Extra-pulmonary tuberculosis, n (%)		129 (22.5)	6 (11.3)	123 (23.7)
	Meningitis	1 (0.2)	1 (1.9)	0 (0)
	Pleural	72 (12.6)	2 (3.8)	70 (13.5)
	Miliary	19 (3.3)	1 (1.9)	18 (3.5)
	Lymph node	25 (4.4)	1 (1.9)	24 (4.6)
	Abdominal	5 (0.9)	1 (1.9)	4 (0.8)
	Pericardial	2 (0.3)	0 (0)	2 (0.4)
	Bones	2 (0.3)	0 (0)	2 (0.4)
	Disseminated	3 (0.5)	0 (0)	3 (0.6)
Laboratory parameters				
	CD4+T (cells/mm^3^), median [IQR]	89 [43-147]	63 [38-111]	92 [44-150]
	HIV-1 RNA, log_10_copies/mL (median, IQR)	5.6 [5.1-6.1]	5.8 [5.3-6.3]	5.6 [5.1-6.1]
	Leucocytes (cells/mm^3^), median [IQR]	3.5 [2.6-4.6]	3.0 [2.7-3.9]	3.6 [2.6-4.7]
	Platelets (cells/uL), median [IQR]	267 [208-343]	275 [211-350]	267 [208-339]
	Haemoglobin (g/dL), median [IQR]	9.4 [8.4-10.3]	9.2 [8.4-9.9]	9.4 [8.4-10.4]
	Antigen HBs reactive, n (%)	122 (21.3)	10 (18.9)	112 (21.5)
	Anti-HBc reactive, n (%)	310 (54.1)	26 (49.1)	284 (54.6)
	Anti-HCV positive*, n (%)	9/539 (1.7)	0/52 (0)	9/487 (1.8)

HbS – Hepatitis B Surface Antigen, HCV – hepatitis C virus, HBV – Hepatitis B Virus

IQR: interquartile range

After exclusion of missing and indeterminate result

### Characterization of TB-IRIS and associated risk factors

Of 573 patients, 118 (20.6%) had a suspicion of TB-IRIS within 12 weeks of starting ART and 53 (9.2%) were classified as TB-IRIS by the review committee. Twenty nine cases were not classified as TB-IRIS due to the presence of exclusion criteria and 36 cases could not be classified as TB-IRIS because their clinical presentation did not fit the definition of the TB-associated IRIS manifestations according to the INSHI case definitions. The median time between the beginning of TB treatment and ART initiation was 4.9 weeks (IQR 4.4-5.1) both for patients with TB-IRIS and for those without. Among patient baseline characteristics, female sex, lower BMI, smear-positive pulmonary TB, lower CD4 count and higher HIV-1 RNA viral load were independently associated with the occurrence of TB-IRIS (Table 2). 

**Table 2:** Analysis of risk factors for paradoxical TB-IRIS (N=573).

		**Occurrence of paradoxical TB-IRIS**				
		%	Unadjusted OR (95% CI)	P	Adjusted OR (95% CI)	P
Age (years)			0.97 (0.93-1.00)	0.051		
Sex						
	Male	7.2	1	0.052	1	
	Female	12.0	1.76 (0.99-3.10)		2.19 (1.21-3.96)	0.0094
Body mass index (kg/m^2^)			0.87 (0.77-0.98)	0.021	0.87 (0.77-0.98)	0.0118
CD4 (by group of 50 cells/mm^3^)			0.75 (0.59-0.95)	0.018	0.77 (0.60-0.99)	0.0395
HIV-1 RNA (log_10_ copies/mL)			1.59 (1.03-2.45)	0.036	1.66 (1.06-2.59)	0.0218
Tuberculosis						
	PTB Smear-negative*	8.0	1		1	
	PTB Smear-positive	13.2	1.73 (0.93-3.23)	0.019	1.92 (1.02-3.64)	0.0227
	Extra-pulmonary TB**	4.7	0.56 (0.22-1.44)		0.66 (0.25-1.74)	
ARV treatment group						
	Nevirapine	11.2	1	0.106		
	Efavirenz	7.3	0.62 (0.35-1.11)			
Time between TB-ART initiation (weeks)			1.27 (0.81-1.98)	0.293		
Hepatitis B						
	Reactive	8.2	1			
	Non reactive	9.2	1.13 (0.55-2.33)	0.731		
Hepatitis C						
	Reactive	9.1	1			
	Non reactive	9.7	1.07 (0.13-8.52)	0.949		

PTB – Pulmonary TB;

^*^ Including patients with pulmonary TB who were unable to produce sputum specimens

^**^ Including 3 patients with disseminated TB

Body mass index, CD4 count and HIV-1 RNA at ART initiation were quantitative variables. The risk of TB-IRIS decreased by 13% with an increase of one Kg/m^2^ of body mass index; by 23% with an increase of 50 CD4 cells /mm^3^ and increased by 66% with an increase of one of HIV-1RNA In 58.5% patients (31/53), the IRIS was an exacerbation of the existing pulmonary TB (27), pleural TB (1), TB meningitis (1), lymph node TB (1) or abdominal TB (1). Twenty-one patients initially diagnosed with pulmonary TB (11 smear-positive and 9 smear-negative) presented an extra-pulmonary TB-IRIS that included ganglionnary (8), ascites (5), meningitis (3), disseminated (2), pleural (1) and arthritis (1) cases. One patient initially diagnosed with pleural TB presented with meningitis TB-IRIS, and one with miliary TB was diagnosed with pulmonary TB-IRIS. Of the 27 exacerbations of pulmonary TB, 26 (96.3%) were defined by the association of two minor clinical criteria.

Median time to diagnosis of TB-IRIS was 21 days (IQR 12; 34). The TB-IRIS occurred within the first 4 weeks after starting ART in 36 patients (67.9%) and between 4 and 12 weeks in 17 patients (32.1%). Occurrence of a new TB lesion in a different localization was more frequent among patients with IRIS occurring after at least 4 weeks (11/17, 64.7%) than among those with earlier IRIS (6/17, 35.3%), p=0.018. Patients with IRIS occurring at 4 weeks or later (6/17, 35.3%) required hospitalization more often than those with IRIS that presented earlier (3/36, 8.3%), p=0.04. Patients’ characteristics, case fatality rate and HIV-1 RNA response at 12 weeks showed no significant differences between the patient group with TB-IRIS occurring at 4 weeks or later and the group with earlier IRIS (Table 3).

**Table 3:** Patients’ characteristics of patients with paradoxical tuberculosis-associated IRIS occurring within first four weeks and between 4 and 12 weeks.

			0-4 weeks N=36	4-12 weeks N=17	p
Age (years), median [IQR]			31 [26-38]	33 [29-38]	0.328
Women n (%)			20 (55.6)	9 (52.9)	0.858
Baseline characteristics					
	Body Mass Index (Kg/m^2^), median [IQR]		18 [16.9-19.3]	17.8 [16.5-18.4]	0.234
	Smear-positive pulmonary tuberculosis, n (%)		21 (58.3)	8 (47.1)	0.441
	Smear-negative pulmonary tuberculosis, n (%)		11 (30.6)	7 (41.2)	0.446
	Extra-pulmonary tuberculosis, n (%)		4 (11.1)	2 (11.8)	0.944
	CD4 (cells/mm^3^), median [IQR]		78 [48-113]	48 [25-95]	0.168
	HIV-1 RNA, log10copies/mL (median, IQR)		5.9 [5.3-6.3]	5.8 [5.4-6.3]	0.430
IRIS n (%)					
	Exacerbation of pulmonary TB		22 (62.9)	5 (29.4)	0.019
		One major sign	1	0	
		Two minor signs	21	5	
	Exacerbation of extra pulmonary TB		3 (8.3)	1 (5.9)	0.809
	Occurrence of new lesion in a different site*		11 (30.5)	11 (64.7)	0.018
	Hospitalisation		3 (8.3)	6 (35.3)	0.040
	Death		3 (8.3)	2 (11.8)	0.917
	Reduction of > 1log10 HIV-1 RNA at week 12		33/33 (100)	13/14 (92.9)	0.121

^*^ 21 cases of new extra-pulmonary IRIS in patients treated for pulmonary TB and 1 case of pulmonary IRIS in a patient initially treated for a miliary TB.

The TB-IRIS resolved in 45/53 (84.9%) patients without adding or stopping any treatment, and in 3 (5.7%) patients after prescription of corticosteroids; in 5 (9.4%) patients it resulted in death. Nine patients (17%) were hospitalized due to TB-IRIS. Three patients died during admission due to disseminated TB-IRIS and 2 died at home following exacerbation of pulmonary TB. None of these deaths occurred in patients who received treatment with corticosteroids. 

### Treatment outcomes and mortality

**Figure 2:**
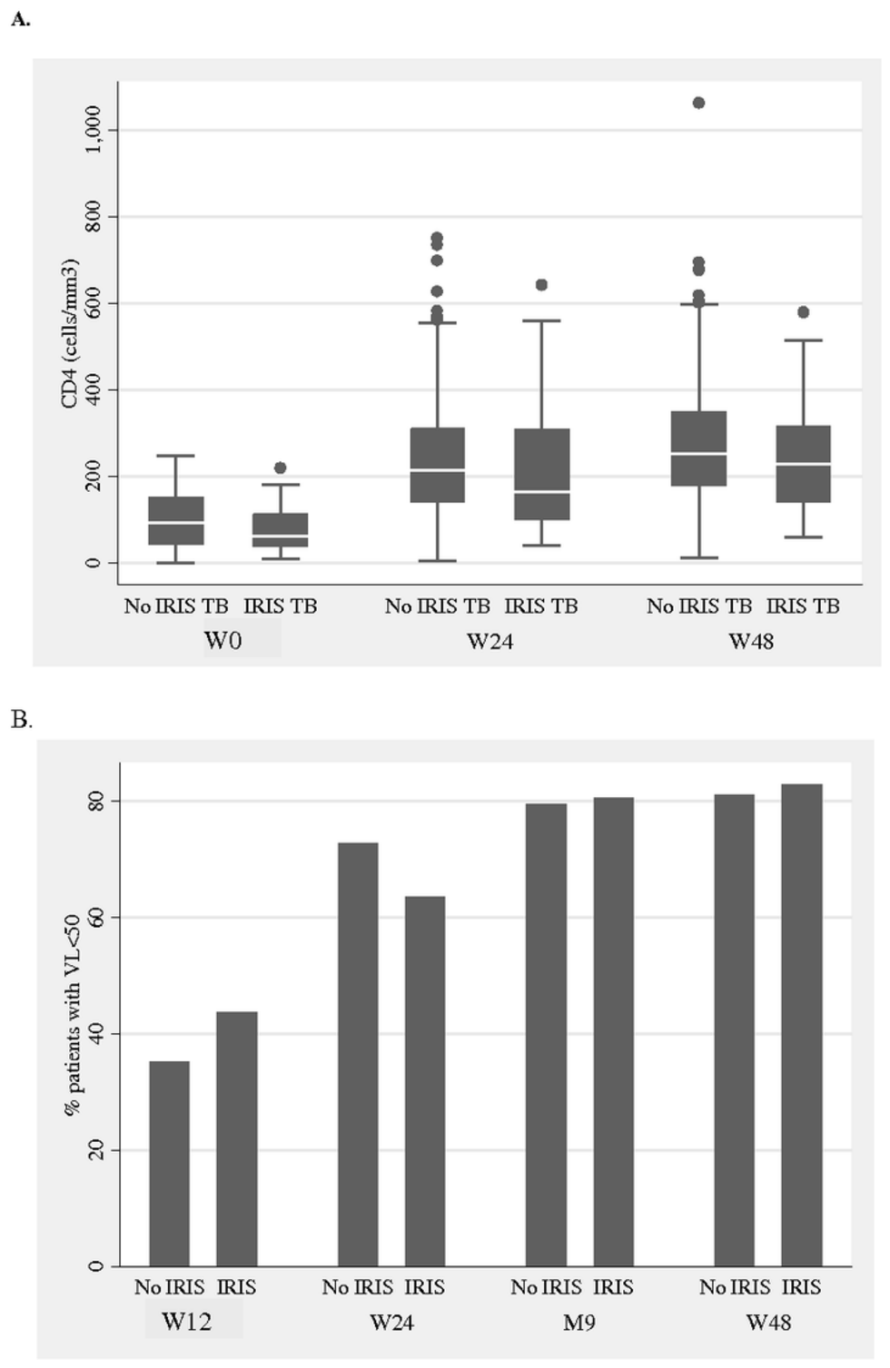
Evolution of CD4 and HIV-1 RNA among patients with and without TB IRIS. A CD4 cell count. B. HIV-1 RNA below 50copies/mL.

There were no significant differences in HIV-1 RNA viral load suppression or CD4 count recovery between patients with and without TB-IRIS (Table 4, Figure 2). TB treatment success rates were also similar between the two groups. Among patients who presented with a TB-IRIS, 3 died secondarily after resolution of the IRIS: one due to a general sepsis (confirmed by autopsy) at week 13, one due to septic shock in a patient treated for an empyema at week 14, and one due to recurrence of miliary TB at week 27.

**Table 4:** Outcomes at 12 weeks post-ART initiation, with and without paradoxical TB-associated IRIS.

			IRIS n (%) N=48	Non IRIS n(%) N=495	p
Deaths			3 (6.3)	11 (2.2)	0.093
HIV response					
	Reduction of > 1 log_10_ HIV-1 RNA at week 12, n (%)		46/47 (97.9)	458/484 (94.6)	0.334
	HIV-1 RNA <50 copies/mL at week 24, n (%)		28/44 (63.6)	338/465 (72.7)	0.202
	HIV-1 RNA <50 copies/mL at week 48, n (%)		34/41 (82.9)	351/433 (81.1)	0.770
	Increase of CD4 (cells/mm^3^) at week 24*, median [IQR]		99 [50-199]	112 [63-186]	0.806
		CD4 > 200 cells/mm^3^ at week 24, n (%)	18/44 (40.9)	246/449 (54.8)	0.078
	Increase of CD4 (cells/mm^3^) at week 48*, median [IQR]		131 [85-221]	156 [91-227]	0.259
		CD4 > 200 cells/mm^3^ at week 48, n (%)	22/39 (56.4)	290/430 (67.4)	0.162
Tuberculosis					
	Treatment success		45 (93.8)	476 (96.2)	0.418
	24 weeks post-treatment success (absence of TB relapse)		44 (91.7)	465 (93.9)	0.976

Overall, 35 patients died after starting ART within the 48-week study follow-up. The median time to death was 7.1 weeks (IQR 2.1, 17.4). Autopsy reports were available for only 6 of these patients. As determined by the available clinical and laboratory data, the causes of deaths were related to: i) TB in 8 patients: MDR-TB (2), disseminated TB (2), poor adherence (1), TB recurrence (2) and severe hemoptysis (1); ii) IRIS in 7 patients: TB-IRIS (5) and IRIS-Kaposi sarcoma (2); iii) AIDS-defining illness in 6 patients: disseminated Kaposi (4) and HIV-related wasting syndrome (2); iv) severe sepsis other than TB in 6 patients: Ludwig’s angina (1), bacterial pericarditis (1), pneumonia (1), cellulitis (1) and septic shock (2); v) other causes in 3 patients: Guillain Barré Syndrome (1), multiple injuries following a car accident (1) and tumor of the hypopharynx with respiratory failure (1). The causes of death were not identified in 5 patients because death occurred either at home or in a health facility outside of Maputo city. 

**Figure 3:**
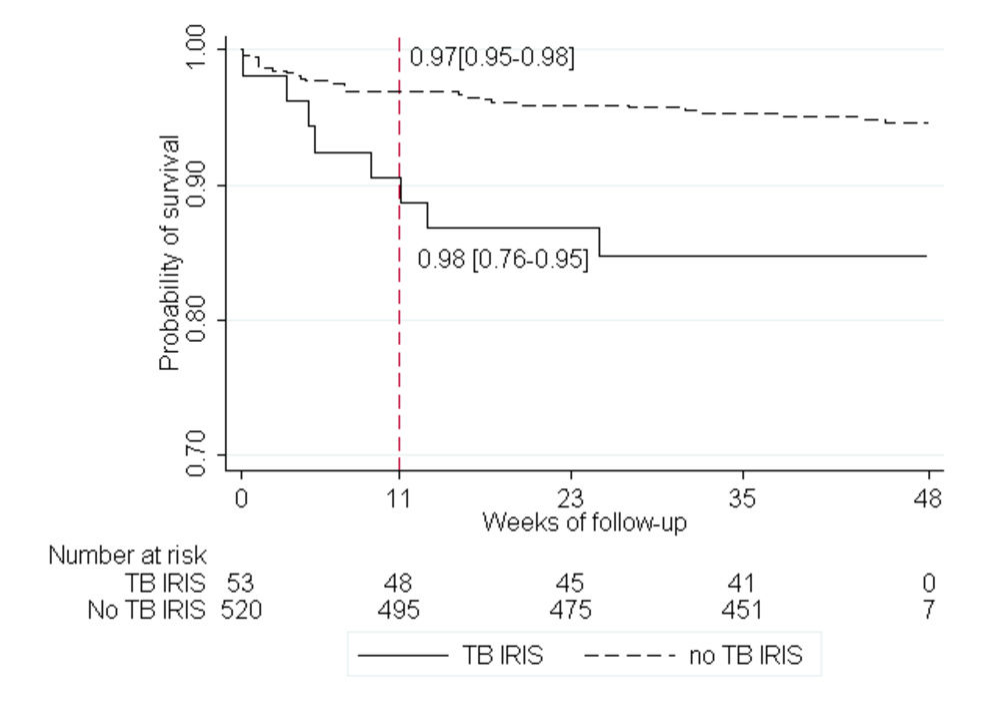
Probability of death among patients with and without TB-IRIS. * Log rank test.

After adjustment for baseline BMI (aOR 1.54, 95%CI 0.99-33.3), CD4 count (aOR 1.54, 95%CI 1.10-2.13) and hemoglobin level (aOR 3.68, 95%CI 1.54-8.76 for hemoglobin between 7 and 9.4g/dL and aOR 4.68, 95%CI 0.84-26.11 for hemoglobin < 7d/dL using hemoglobin > 9.5g/dL as reference), the occurrence of a TB-IRIS was independently associated with 48-week mortality (aOR 2.72, 95%CI 1.14-6.54). The cumulative probabilities of survival at week 12 were 0.89 and 0.97 for patients with and without TB-IRIS, respectively (Figure 3). 

## Discussion

This is one of the largest studies in a sub-Saharan African country to assess both the incidence of paradoxical TB-associated IRIS within 3 months of starting ART and its effect on 48-week outcomes of HIV-TB co-infected patients. The incidence we found was relatively low (9.3%) compared with the 15.7% figure obtained in a recent meta-analysis that evaluated multiple studies on IRIS up to 2009 (95%CI 9.7-24.5)[10]. This might be explained by our study’s use of the more stringent INSHI case definition, which restricted the definition of paradoxical TB-associated IRIS to the 3-month period after ART initiation, in contrast to the previous studies included in the meta-analysis and that had no time-restricted definition. However, our results are in the range of the 4-21% paradoxical TB-associated IRIS incidence reported in 5 more recent studies, including 3 that used the INSHI case definition [16-20]. The incidence in our study is particularly low considering the early ART initiation (median of 5 weeks) and low CD4 count (median 92 cells/mm^3^) of patients at ART initiation. It is possible that the incidence of TB-IRIS may be underestimated in this trial due to the exclusion of TB-HIV co-infected patients with any serious clinical or laboratory sign at ART initiation. Indeed, these patients were likely to be severely immunosuppressed and to present a TB-IRIS. Taking into consideration our use of systematic case reporting for TB-IRIS in this trial, and the retrospective review of all patient files, the underreporting of TB-IRIS is unlikely. 

Differences in clinical presentation between patients with an IRIS during the first 4 weeks of ART and those with a later IRIS have not been previously described. In a study conducted by Worodria et al. in Uganda, there were no differences in TB-IRIS clinical presentation between those cases occurring within the first three months of ART and those occurring later [21]. Unfortunately, due to the low number of TB-IRIS cases in our study, we could not investigate these clinical differences further. Also, the study was not designed to assess the immunopathogenesis that might explain the differences and may deserve further analysis.

In agreement with other studies, we found that lower CD4 count, higher HIV-1 viral load and lower BMI at ART initiation were independently associated with the risk of TB-IRIS [12,19,20]. In addition, our study found an association between TB-IRIS and female sex that has not been reported in the scientific literature and is difficult to explain. It could be potentially indirectly related to the better CD4 cells reconstitution observed in women compared to men [22]. The association between pathogen burden and the risk of IRIS has been raised previously but very few studies specifically assessed the effect of *Mycobacterium tuberculosis* burden [23,24]. Our study’s exclusion of patients with serious clinical symptoms at ART initiation may explain the absence of an association between disseminated TB and TB-IRIS, as noted in some previous reports [11,25,26]; our study cohort had only three patients with disseminated TB. The absence of an association between TB-IRIS occurrence and time from TB treatment start to ART initiation probably reflects the fact that our trial protocol was to start all patients on ART at 4-6 weeks after TB treatment initiation. Despite the fact that the same pro-inflammatory chemokines and cytokines, such as the natural killer cell chemoattractant CXCL10, are elevated after ART initiation in both TB-IRIS and hepatitis flares in patients co-infected with HIV and hepatitis B virus (HBV), and that 20% of our study population was co-infected with HBV, we did not find any association between HBV co-infection and the occurrence of TB-IRIS [24]. 

Despite the high rate of patient follow-up (due to favorable trial conditions, as evidenced by a low overall mortality (6.1%)), the case fatality rate of TB-IRIS was relatively high (9.4%) compared to the average of 3.2% (95%CI 0.7-9.2) reported in the latest meta-analysis[10]. However, it was close to the case fatality rate (10%) reported in a more recent prospective cohort study in Uganda [21]. This high rate might be explained by the underuse of corticosteroids in our study [27,28]; indeed, none of the five patients who died due to TB-IRIS received corticosteroids. On the other hand, any paradoxical IRIS associated to TB or other pathogens accounted for 20% of the total number of deaths in our study, similar to what has been previously reported [10,29]. 

The occurrence of TB-IRIS was independently associated with the 48-week post-ART mortality after adjustment for patients’ BMI, CD4 count, HIV-1 RNA viral load and hemoglobin. To our knowledge, this has not been reported previously. Although not significant, a trend of higher mortality was reported in Thailand among patients with TB-IRIS (9.5%) compared to patients without IRIS (2.1%) but the comparison was not adjusted with the baseline patient characteristics, due to the small sample size [30]. On the other hand, an association between the occurrence of any IRIS and the risk of all-cause mortality after adjusting for baseline CD4 was reported in a small and in a very large retrospective cohort study conducted in Mexico and in the United States, respectively [31,32]. However, in these studies the majority of IRIS were unmasking IRIS, which make difficult to distinguish from the occurrence of an opportunistic infection. This is particularly the case in the large cohort study from the United States, which defined any Type B and C illnesses occurring between 7 and 180 days after ART initiation as IRIS. Furthermore, unmasking TB-IRIS and paradoxical TB-associated IRIS have different clinical presentation and pathogenesis and therefore most likely different clinical outcomes. In our study, this association might just reflect the high case fatality rate of TB-IRIS in the 3 months after starting ART.

Interestingly, we did not detect any difference between short (12 weeks) and long term (48 weeks) HIV-1 RNA viral load suppression in patients with and without TB-IRIS. Similarly, CD4 recovery and TB treatment outcomes did not differ between the two groups. This is consistent with what has been reported in a recent study in India [33]. However, this might be biased by the fact that patients with TB-IRIS had a higher case fatality rate. 

The main limitations of the study were related to the design of the original CARINEMO trial. The trial did not include all HIV-TB co-infected patients, especially the more severe cases, which may have introduced a selection bias. The time interval for initiating ART after starting TB treatment was restricted, which in turn affected the analysis between time to initiate ART and TB-IRIS occurrence. Although the use of the INSHI case definition improves the comparability among studies, it probably contributed to underestimating the true incidence of paradoxical TB-associated IRIS in our study population. 

In conclusion, in this large cohort of HIV-TB co-infected patients on ART, the occurrence of a paradoxical TB-associated IRIS within 12 weeks after ART initiation was independently associated with the 48-week mortality of HIV-TB co-infected patients. Although this unexpected outcome might be explained by the high case fatality rate of TB-IRIS in this study, it may still justify further assessment in large prospective cohorts. On the other hand, the 48-week CD4 recovery, virological suppression and TB treatment outcomes did not seem to be affected by the occurrence of a TB-IRIS.
