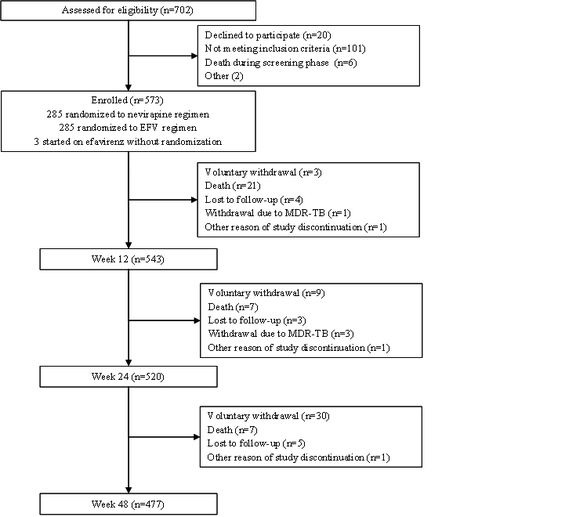# Correction: Incidence of Paradoxical Tuberculosis-Associated Immune Reconstitution Inflammatory Syndrome and Impact on Patient Outcome

**DOI:** 10.1371/annotation/15d01128-2495-4b95-8fe8-8702190fdb0e

**Published:** 2014-01-31

**Authors:** Maryline Bonnet, Elisabeth Baudin, Ilesh V. Jani, Elizabete Nunes, François Verhoustraten, Alexandra Calmy, Rui Bastos, Nilesh B. Bhatt, Christophe Michon

The incorrect Figure 1 appears in the online version of this article. The correct Figure 1 can be viewed here: 

**Figure:**